# Insecta

**Published:** 1840-04

**Authors:** 


Art. IV.—*Insecta.* By George Newport, Esq. (*The Cyclopædia of Anatomy and Physiology.*)—*London,* 1839. 8vo, pp. 128.

The work of which this article forms no unimportant part continues to advance regularly though slowly; but we shall not complain of this, so long as the period of delay is so well occupied as it must have been in the preparation of this most elaborate essay. It is one which does credit to the judgment of the editor in the selection of the writer, as well as to the author in the combination and arrangement of his materials. We much question if any country could furnish a naturalist better qualified for such a task than Mr. Newport. To unwearied zeal in his pursuit, he unites great powers of observation, and sagacity in the employment of opportunities of research. He never allows himself to be led astray by theory, but honestly states facts. A very large proportion of this article consists of original matter; and the remainder is drawn from the most authentic sources. After a general view of the position of insects in the animal scale, an outline of their classification is given. The different states of the insect—larva, pupa, and imago—are then noticed, as they present themselves in the chief subdivisions of the group; and the principal phenomena in the life of each are detailed. The external anatomy of the perfect insect is then described with great minuteness; we should say too minutely for the character of the work. Details of this kind are of great importance to the naturalist, but of much less consequence to the comparative anatomist, and of still less to the physiologist. The muscular and nervous systems are then treated of; and the changes which the latter undergoes during the metamorphosis are exhibited. The organs of nutrition, secretion, and reproduction, with their respective functions, then come under review. We could have wished that the author had been a little fuller on some parts of this division, and had curtailed the former. We should have liked to have seen a general account of the food of insects, and of the functions of the group in the economy of nature; and, under the head of reproduction, some details relative to the extraordinary increase in number occasionally observed. However, we receive with much thankfulness what we have got; and we hope that Mr. Newport will long continue to prosecute his labours with the skill and enthusiasm which he here displays.

We cannot dismiss this notice without once more formally calling the earnest attention of our readers to the Cyclopædia of Anatomy and Physiology. It continues to possess the great excellence which it manifested at its commencement, and is, indeed, a work of unequalled value to the physiologist and comparative anatomist.

